# Morphometric Analysis of Cranial Shape in Fossil and Recent Euprimates

**DOI:** 10.1155/2012/478903

**Published:** 2012-05-07

**Authors:** C. Verity Bennett, Anjali Goswami

**Affiliations:** ^1^Department of Genetics, Evolution and Environment, University College London, London WC1E 6BT, UK; ^2^Department of Earth Sciences, University College London, London WC1E 6BT, UK

## Abstract

Quantitative analysis of morphology allows for identification of subtle evolutionary patterns or convergences in anatomy that can aid ecological reconstructions of extinct taxa. This study explores diversity and convergence in cranial morphology across living and fossil primates using geometric morphometrics. 33 3D landmarks were gathered from 34 genera of euprimates (382 specimens), including the Eocene adapiforms *Adapis* and *Leptadapis* and Quaternary lemurs *Archaeolemur*, *Palaeopropithecus*, and *Megaladapis*. Landmark data was treated with Procrustes superimposition to remove all nonshape differences and then subjected to principal components analysis and linear discriminant function analysis. Haplorhines and strepsirrhines were well separated in morphospace along the major components of variation, largely reflecting differences in relative skull length and width and facial depth. Most adapiforms fell within or close to strepsirrhine space, while Quaternary lemurs deviated from extant strepsirrhines, either exploring new regions of morphospace or converging on haplorhines. Fossil taxa significantly increased the area of morphospace occupied by strepsirrhines. However, recent haplorhines showed significantly greater cranial disparity than strepsirrhines, even with the inclusion of the unusual Quaternary lemurs, demonstrating that differences in primate cranial disparity are likely real and not simply an artefact of recent megafaunal extinctions.

## 1. Introduction

Euprimates comprises two principal sister groups: Strepsirrhini, including Lemuriformes and Lorisiformes; and Haplorhini, including Tarsiiformes and Simiiformes (Anthropoidea). Strepsirrhines have a smaller geographic range, occupying parts of Southern Africa, Madagascar, and Southeast Asia, than do haplorhines, which, excluding humans, occupy every continent except Australia and Antarctica. In addition, haplorhine primates are far more speciose (~300 species) than strepsirrhines (~100 species) [1]. Tarsiiformes have previously been grouped with the strepsirrhines as “prosimians”, but most recent molecular and morphological analyses [2–4] have placed them firmly within Haplorhini (but see [5]).

Estimates of the time of divergence of strepsirrhines and haplorhines are heavily debated. The earliest euprimate currently recognised is the late Paleocene *Altiatlasius *[6, 7]. However, molecular clock models and statistical models based on fossil occurrences place the origin of Euprimates between 70–103 mya (million years ago), well into the Cretaceous [8], despite the lack of any unambiguous placental mammal fossils prior to 64 mya [9].

Within Haplorhini, the first undisputed anthropoids are known from the late middle Eocene of Libya [10], although taxa from the early and middle Eocene of Asia [11–14], as well as *Altiatlasius* from the late Paleocene of North Africa [15], have also been tentatively assigned to Anthropoidea. The paucity of anthropoid fossils from the early and middle Eocene makes it impossible to reasonably infer their continent of origin and the nature of their early dispersal, although there is some support for an African or Indo-Madagascan origin [16, 17].

The sister group to anthropoids, Tarsiiformes, has an exceedingly sparse fossil record. Other than the extant genus, *Tarsius*, only a few other genera are known, and only one (*Xanthorhysis* from the middle Eocene of China) is uncontentious [18–20]. Nonetheless, there is evidence that even the earliest tarsiers had greatly enlarged orbits, similar to extant species [18].

The strepsirrhine fossil record also begins in the Eocene, but, unlike haplorhines, crown strepsirrhine fossils are restricted to Africa and Madagascar. These early forms appear most similar to extant lorises and galagos, which are reconstructed as having an Afro-Arabian origin in the late middle Eocene [21]. Among the most interesting aspects of strepsirrhine evolution is the adaptive radiation of lemurs on Madagascar, estimated by molecular clocks to have begun between 62–54 million years ago (Ma) [22–24]. Madagascan lemurs represent an endemic and monophyletic radiation that dispersed via rafting from mainland Africa to Madagascar, where the availability of unoccupied niches led to an opportunity for rapid diversification of the group [22]. At least 15 lemur species, including all of the large-bodied forms such as *Megaladapis*, have gone extinct during the Holocene. As the demise of these species follow human colonisation of Madagascar, with some last occurrences as recent as ~500 years ago, there is ongoing debate about the role of humans in causing these extinctions [25, 26].

In addition to the extant clades of strepsirrhines and haplorhines, two extinct groups of euprimates radiated across North America, Europe, Asia, and North Africa during the Eocene: Adapiformes and Omomyiformes. Omomyiformes is a taxonomically diverse group of small-bodied, predominantly insectivorous primates that is typically allied with haplorhines and may be more closely related to tarsiers than to anthropoids within haplorhini [27]. Adapiformes is also taxonomically diverse, with at least seven subfamilies and 30 genera that show great variation in dental and cranial morphology and locomotor specialisations of the postcranial skeleton [4, 28]. Whether this group is more closely related to Haplorhini or to Strepsirrhini has been heavily debated for example, [4, 29], but the most recent cladistic analyses support strepsirrhine affinities for adapiforms [30].

Primates vary in habitat preference (tropical and temperate, arboreal, and terrestrial), diet (folivorous, frugivorous, insectivorous, gummivores, and carnivorous), and even in temporal niche (nocturnal, diurnal, and cathemeral). This breadth of ecological range is reflected in the diversity of cranial morphologies across Primates. Numerous studies have quantified aspects of morphological evolution in the primate skull, but these are mostly focused on a specific clade within Primates (e.g., [31–34]) or on a specific component of the cranium across a broader range of taxa (e.g., [35, 36]). Morphometric analysis of cranial shape allows for identification of subtle patterns or convergences in anatomical evolution that can aid ecological interpretations of extinct taxa. However, to date only a single study has quantified cranial morphology across all extant primates [37]. Even more strikingly, given the broad interest in the fossil record of primates, no study has yet included fossil forms within such a framework.

Here, we present 3D morphometric analysis of a broad sample of extant euprimates and well-preserved (un-deformed) fossil euprimates, including 2 adapiform genera and 3 genera of Quaternary lemurs. We assess whether these fossil taxa explored different regions of morphospace to their extant relatives, identify shape differences that distinguish the major clades, and discuss convergences across clades that may reflect ecological similarities.

## 2. Methods

**Figure 1:**
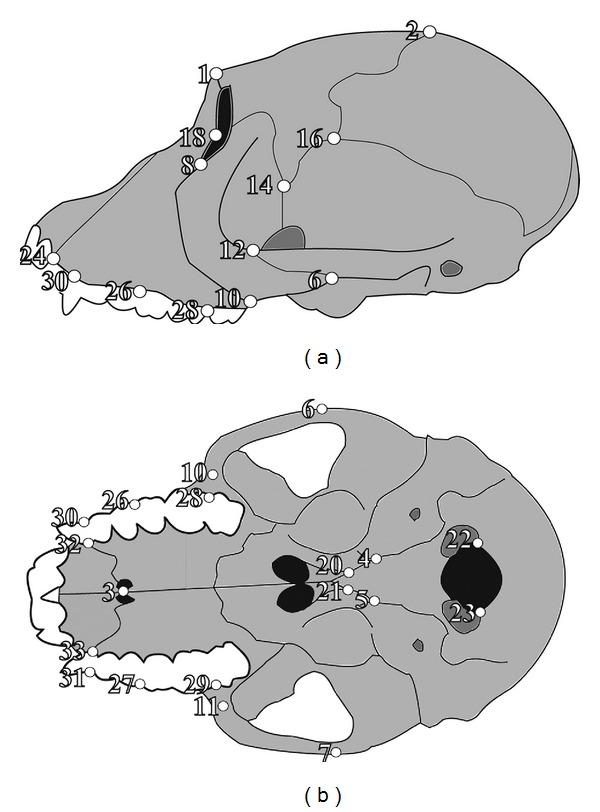
Location of cranial landmarks (white circles) as viewed (a) laterally and (b) ventrally. Numbers correspond to landmarks as listed in Table 1.

33 cranial landmarks (Table 1, Figure 1) were collected from 29 extant genera and 5 fossil genera of euprimates (Table 2) using a MicroScribe G2X digitiser with a reported accuracy of 0.23 mm. Due to the incomplete nature of fossil material, bilateral landmarks missing from either the right or left side of the skull were mirrored about the midline in Mathematica 6.0 (Wolfram Research Inc., Champaign, IL) in order to maximise sample sizes. Data was subjected to Procrustes superimposition to remove nonshape information [38], and then principal components analysis (PCA) was performed to analyse morphological difference between individuals [39]. A linear discriminant function analysis (linear DFA) was performed in a pairwise fashion between the following groups of primates: Anthropoidea, Tarsiiformes, extant Lemuriformes, fossil Lemuriformes, all fossil and extant Lemuriformes, Lorisiformes, and Adapiformes. A linear DFA was also performed, using MorphoJ [40], between Strepsirrhini and Haplorhini with Adapiformes included first in the former and then the latter, to more robustly test for morphological similarity, given the small adapiform sample size. To reduce sampling effects, data was pooled by species for each analysis. To account for allometric effects on cranial shape, a PCA was also performed on the residuals of a multivariate regression of shape with centroid size. Prior to correction for allometry, an analysis of disparity, measured as variance, was calculated for recent haplorhines, recent strepsirrhines, and recent strepsirrhines plus Quaternary lemurs using the Simple3D program in IMP7 [41].

## 3. Results

### 3.1. Principal Components Analysis

**Figure 2:**
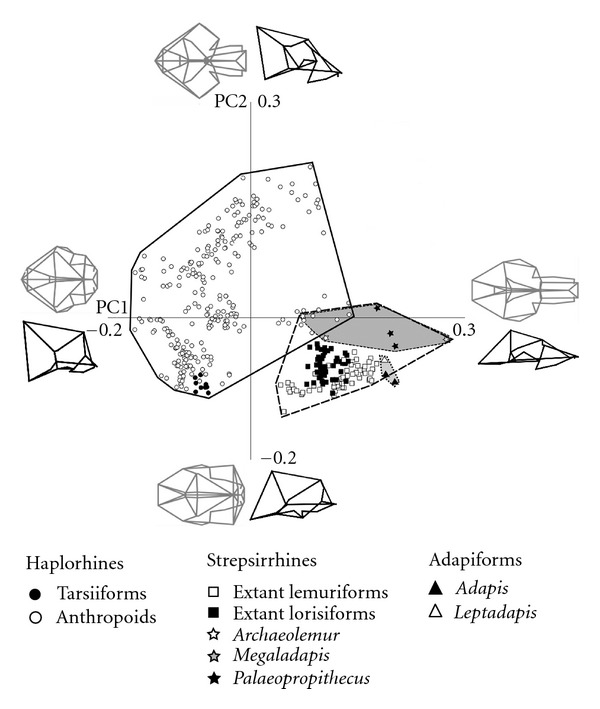
Principal components 1 and 2, with wireframes of cranial shapes represented at positive and negative extremes of each axis in dorsal (grey) and lateral (black) views.

PC1 accounted for 29% of the total variance in the dataset prior to correction for allometry. This axis divided haplorhines, with laterally wide, dorsoventrally tall skulls and flat faces, at the negative end from strepsirrhines, with laterally narrower, dorsoventrally shorter skulls, elongate faces, and narrow snouts, at the positive end (Figure 2). The basicranium was also more horizontal at the positive end of PC1 and more inclined at the negative end. *Megaladapis *defined the positive extreme of this axis, while howler monkeys and *Archaeolemur* overlapped in the middle. All fossil taxa, including adapiforms and Quaternary lemurs, fell in the positive end of PC1.

**Figure 3:**
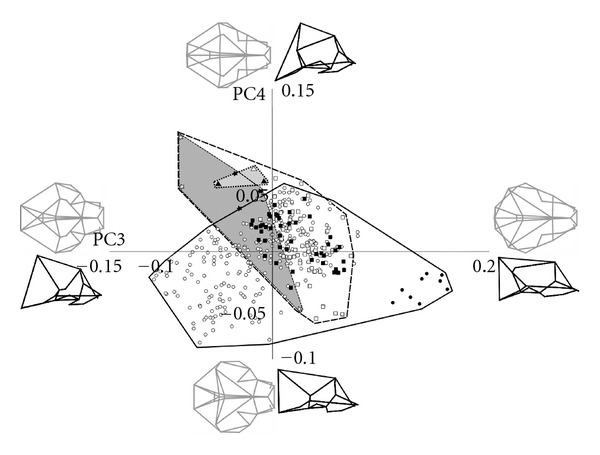
Principal components 3 and 4 with wireframes of cranial shapes represented at positive and negative extremes of each axis in dorsal (grey) and lateral (black) views. Symbols as shown in Figure 2.

PC2 accounted for 16% of the variance (Figure 2) and involved a shift from a narrow-faced skull with an anteroposteriorly shorter neurocranium at the positive end, to a very wide face with a longer neurocranium at the negative end. The tooth row also fell below the ventral extent of the basicranium at the positive extreme of PC2 whilst at the negative extreme, occupied by extant strepsirrhines and fossil adapiforms, the tooth row was higher. Tarsiers also fell toward the negative end of this axis whilst anthropoid primates were distributed across the full range of PC2. PC3 accounted for 6.5% of the variance and showed dorsoventrally shorter, wider faced skulls with more vertical posterior vaults at the positive end and dorsoventrally taller skulls with narrower faces, and more gently sloping vaults at the posterior (Figure 3). This axis separated tarsiers, which plot furthest towards the positive end than any other group, from the rest of the haplorhine primates. PC4 accounts for 5.1% of the variance and largely showed a shift from laterally narrower skulls with the basicranium falling more ventrally than the tooth row at the positive end to laterally wider skulls with a flat posterior vault at the negative extreme. *Archaeolemur *and the fossil adapiforms fell towards the positive end of this axis.

Strepsirrhines and haplorhines were well separated along the first two principal components of variation, with the exception of a small area of overlap between the haplorhine genus *Alouatta *(howler monkeys) with extant strepsirrhines and *Archaeolemur*. There was a much greater area of overlap between the two groups on PCs 3 and 4, although, in contrast to the morphospace occupied in PCs1 and 2, *Archaeolemur* plotted further away from haplorhines than the other strepsirrhines did.

**Figure 4:**
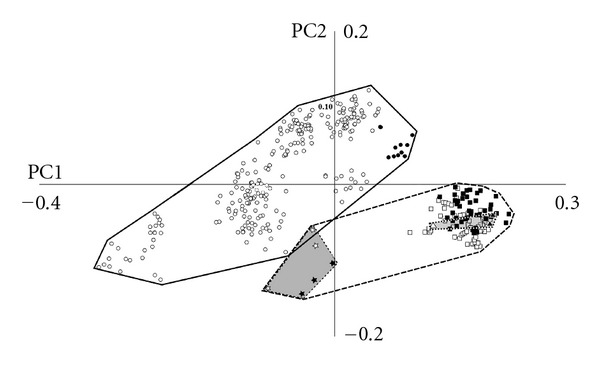
Principal components 1 and 2 after the removal of allometric effects. Symbols as shown in Figure 2.

After allometry had been corrected for (Figure 4), the first four PCs accounted for 75.8% of the total variation, 19.2% more so than in the uncorrected PCA. PC1 accounted for 52.7% of the variance, PC2 accounted for 12%, PC3 accounted for 7.3%, and PC 4 accounted for 4.8%. The overall distribution of taxa in morphospace and the shape changes associated with each principal component was similar in both the corrected and uncorrected analyses. As in the uncorrected analysis, the adapiforms fell well within the range of recent strepsirrhines, whereas the Quaternary Madagascan lemurs plotted even further outside of the recent strepsirrhine space. Overall, it does not appear that allometry strongly influenced the patterns of morphological disparity at the scale investigated here.

As is clear in Figure 2, haplorhines occupied a larger region of morphospace than strepsirrhines. Correspondingly, disparity, measured as variance, was significantly greater in recent haplorhines (0.0293) than in recent strepsirrhines (0.0116) at *P* < 0.01 significance level. Inclusion of the Quaternary lemurs in the strepsirrhine group significantly increased their disparity (0.0115 to 0.0141, *P* < 0.01), although this expanded strepsirrhine dataset still showed significantly lower disparity than observed in recent haplorhines (*P* < 0.01).

### 3.2. Linear Discriminant Function Analyses

When distances between groups were compared on a pairwise basis by linear DFA, there was no significant distance between adapiforms and lorisiformes or tarsiers. Fossil lemurs were also not significantly separated from adapiforms (*P* < 0.001) but were separated from extant lemuriforms, with a Mahalanobis distance of 53.17 (*P* < 0.001), as well as from lorisiforms, with a Mahalanobis distance of 96.85 (*P* = 0.02). All other between group distances were significant at the *P* < 0.001 significance level.

**Figure 5:**
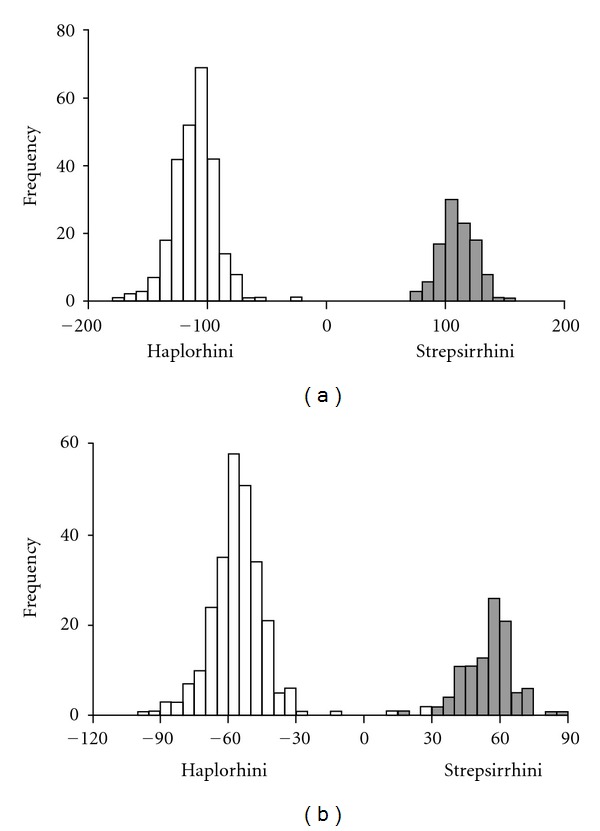
Cross-validation results of the linear discriminant function analysis for strepsirhines (grey) versus haplorhines (white) with (a) adapiforms included with strepsirrhines and (b) adapiforms included with haplorhines.

Two additional linear DFAs were performed between haplorhines and strepsirrhines with adapiforms included first in one group and then in the other (Figure 5). When adapiforms were placed with haplorhines (Figure 5(b)), the Mahalanobis distance between the groups was 10.58, compared to 14.87 when adapiforms were placed with strepsirrhines. When the adapiforms were included within strepsirrhines, cross-validation of the linear DFA results correctly assigned 100% of specimens to their preassigned groups. In contrast, when the adapiforms were included with the haplorhines, cross-validation of the linear DFA results reassigned four specimens from the preassigned haplorhine group to the strepsirrhine group.

## 4. Discussion

Cranial landmarks in this study differ from those of Fleagle et al. [37]. Most notably we used fewer points around the orbit and at the anterior extreme of the skull, as these areas are easily broken in fossil specimens. Despite the different cranial landmarks used here, our results are largely in agreement with those of Fleagle et al. [37] with respect to the general distribution of extant groups in morphospace. For example, both studies found that haplorhines and strepsirrhines were well separated in morphospace with the major components of variation reflecting relative skull length and width and facial depth.

Whereas extant strepsirrhines and haplorhines were entirely separated in morphospace, our results demonstrate that there was greater convergence among these groups in the recent past. Specifically, the Quaternary lemur *Archaeolemur *overlapped with *Alouatta, *the howler monkey (Figure 2), reflecting *Alouatta*'s unusually narrow face (for a haplorhine). Although the overlap with *Alouatta* was weaker after correction for allometric effects, this fossil lemur remained the only strepsirrhine in this sample to invade haplorhine morphospace. It was expected that, after correcting for allometry, *Megaladapis,* the large “koala lemur”, would plot more closely to the space occupied by the other lemuriforms. However, the opposite was observed as this taxon, along with all other fossil lemurs, plotted further away from recent strepsirrhines and closer to haplorhines in the negative realm of PC1 and 2.

Consistent with our results, Fleagle et al. [37] found that, among anthropoids, *Alouatta *plotted closest to the strepsirrhines. They also found that *Tarsier *plots close to the strepsirrhines than other anthropoids do. In this study tarsiers fell well within haplorhine space, although at the “strepsirrhine” end of the haplorhine range on PC2 in the uncorrected analysis (Figure 2) and on PC1 of the corrected analysis (Figure 4). This difference could be due to the difference in landmark distribution across the skull in the two studies, as the orbital region, for which our study has fewer landmarks than the Fleagle et al. [37] study, is particularly unusual in tarsiers. Interestingly, the linear DFA results found significant differences between tarsiiforms and all groups of extant taxa (extant lemuriforms, lorisiforms, and anthropoids). The lack of significant differences between tarsiiforms and the extinct groups (adapiforms or extinct Madagascan lemurs) is likely due to low sample sizes rather than morphological similarity, given their wide separation in morphospace.

The marked separation of strepsirrhines and haplorhines in morphospace indicates that skulls of fossil lemurs, whilst clearly distinct in skull shape relative to extant lemurs, still share key morphological similarities with their closest extant relatives to the exclusion of haplorhines. Nonetheless, their deviance from the range of extant lemuriform skull shape, as demonstrated by the results of both PCA and linear DFA, was evident in the significant increase in the variance of strepsirrhine crania when fossil lemurs were included within this group, discussed further below. The results of the linear DFA also have implications for the affinities of Adapiformes. It is clear that the adapiforms are more similar in their morphology to strepsirrhine rather than haplorhine primates from the shorter distance between the two groups and from the reassignment of specimens upon cross-validation when adapiforms were placed with haplorhines rather than strepsirrhines (Figure 5). Linear DFA is known to be susceptible to sample size effects, so these results are treated with caution here. Moreover, morphological similarity (i.e., phonetic similarity) should not be treated as evidence of phylogenetic relationship. However, the data presented here may prove useful in considering the competing hypotheses for adapiform relationships [4, 29, 30] and may inform character selection or distribution in future phylogenetic analyses.

Lastly, the unequal diversification of sister clades has been a topic of interest for decades, with the marked difference in diversity between recent strepsirrhines and haplorhines being a notable example. The relatively low variance displayed by extant strepsirrhines was indeed expanded significantly by the inclusion of fossil forms, as postulated by Fleagle et al. [37]. However, this variance is still much smaller than that observed in just the extant haplorhines. Of course, there are many species of subfossil lemurs that were not possible to include in this analysis, and additional sampling may further increase the morphospace occupied by living and extinct strepsirrhines.

Moreover, all of the fossils included here are either undoubted strepsirrhines, in the case of the Quaternary lemurs or possible stem haplorhines of debated affinities (adapiforms). Therefore, another promising avenue of future research would be to include fossil crown haplorhines, which could provide important information on the roots of the differential diversity observed in these two clades. Inclusion of fossil haplorhines would also provide data on whether extinct forms were exploring entirely novel regions of morphospace unseen in extant forms, as was observed here for fossil strepsirrhines. Inclusion of a wider range of fossil primate taxa would also increase our understanding of the evolution of cranial diversity with respect to extant groups, particularly with respect to testing hypotheses of rates, constraints, and plasticity within different lineages. Unfortunately, the extent to which this can be achieved is greatly limited by the availability of undeformed and sufficiently complete fossil skulls, a problem which can only be solved by the continued collection effort by palaeontologists in the field.

## Figures and Tables

**Table 1:** Cranial landmarks used in this study. Numbers correspond to those illustrated in Figure 1.

Midline landmarks	
1	Nasal-frontal suture
2	Parietal-frontal suture
3	Premaxilla-maxilla suture

Bilateral landmarks (left and right)	

4 and 5	Basioccipital-basisphenoid-bulla suture
6 and 7	Jugal-squamosal ventral suture
8 and 9	Jugal-maxilla (orbit crest) suture
10 and 11	Jugal-maxilla (base of zygomatic arch) suture
12 and 13	Jugal-squamosal dorsal suture
14 and 15	Frontal-squamosal-alisphenoid suture
16 and 17	Parietal-frontal-squamosal suture
18 and 19	Lacrimal-frontal-maxilla suture
20 and 21	Basisphenoid-presphenoid suture
22 and 23	Occipital condyle extreme
24 and 25	Premaxilla-maxilla lateral suture
26 and 27	Anterior lateral M1
28 and 29	Posterior lateral M2
30 and 31	Canine, lateral extreme
32 and 33	Canine, mesial extreme

**Table 2:** List of species included in the analysis, ^†^ indicates fossil species.

Haplorhini	Strepsirrhini
Simiiformes (Anthropoidea)	Lorisiformes
*Alouatta seniculus*	*Loris tardigradus*
*Aotus trivirgatus*	*Nycticebus coucang*
*Ateles paniscus*	*Perodicticus potto*
*Cacajao calvus*	
*Callicebus moloch*	Lemuriformes
*Callimico goeldii*	*Archaeolemur majori ^†^*
*Callithrix jacchus*	*Avahi laniger*
*Chlorocebus aethiops*	*Eulemur rufus*
*Gorilla gorilla*	*Indri indri*
*Hylobates muelleri*	*Lemur catta*
*Lagothrix lagotricha*	*Lepilemur microdon*
*Lophocebus albigena*	*Megaladapis edwardsi ^†^*
*Macaca fascicularis*	*Paleopropithecus maximus ^†^*
*Miopithecus talapoin*	
*Nasalis larvatus*	Adapiformes*^†^*
*Pan troglodytes*	*Leptadapis magnus ^†^*
*Piliocolobus rufomitratus*	*Adapis parisiensis^†^*
*Saguinus fuscicollis*	
*Saimiri sciureus*	
*Trachypithecus phayrei*	
	
Tarsiiformes	
*Tarsius syrichta*	

## References

[1] Wilson DE, Reeder DM (2005). *Mammal Species of the World. A Taxonomic and Geographic Reference*.

[2] Poux C, Douzery EJP (2004). Primate phylogeny, evolutionary rate variations, and divergence times: a contribution from the nuclear gene IRBP. *American Journal of Physical Anthropology*.

[3] Schmitz J, Ohme M, Zischler H (2001). SINE insertions in cladistic analyses and the phylogenetic affiliations of Tarsius bancanus to other primates. *Genetics*.

[4] Seiffert ER, Perry JMG, Simons EL, Boyer DM (2009). Convergent evolution of anthropoid-like adaptations in Eocene adapiform primates. *Nature*.

[5] Chatterjee HJ, Ho SY, Barnes I, Groves C (2009). Estimating the phylogeny and divergence times of primates using a supermatrix approach. *BMC Evolutionary Biology*.

[6] Bloch JI, Silcox MT, Boyer DM, Sargis EJ (2007). New Paleocene skeletons and the relationship of plesiadapiforms to crown-clade primates. *Proceedings of the National Academy of Sciences of the United States of America*.

[7] Seiffert ER, Simons EL, Clyde WC (2005). Basal anthropoids from Egypt and the antiquity of Africa’s higher primate radiation. *Science*.

[8] Wilkinson RD, Steiper ME, Soligo C, Martin RD, Yang Z, Tavaré S (2011). Dating primate divergences through an integrated analysis of palaeontological and molecular data. *Systematic Biology*.

[9] Goswami A, Prasad GVR, Upchurch P (2011). A radiation of arboreal basal eutherian mammals beginning in the Late Cretaceous of India. *Proceedings of the National Academy of Sciences of the United States of America*.

[10] Jaeger JJ, Beard KC, Chaimanee Y (2010). Late middle Eocene epoch of Libya yields earliest known radiation of African anthropoids. *Nature*.

[11] Jaeger JJ, Thein T, Benammi M (1999). A new primate from the Middle Eocene of Myanmar and the Asian early origin of anthropoids. *Science*.

[12] Beard KC, Qi T, Dawson MR, Wang B, Li C (1994). A diverse new primate fauna from middle Eocene fissure-fillings in southeastern China. *Nature*.

[13] Bajpai S, Kay RF, Williams BA, Das DP, Kapur VV, Tiwari BN (2008). The oldest Asian record of Anthropoidea. *Proceedings of the National Academy of Sciences of the United States of America*.

[14] Rose KD, Rana RS, Sahni A (2009). Early eocene primates from Gujarat, India. *Journal of Human Evolution*.

[15] Sigé B, Jaeger J, Sudre J, Vianey-Liaud M (1990). *Altiatlasius koulchii*n. gen. et sp., primate omomyidé du Paléocène supérieur du Maroc, et les origines des euprimates. *Palaeontographica Abteilung A*.

[16] Miller ER, Gunnell GF, Martin RD (2005). Deep time and the search for anthropoid origins. *American Journal of Physical Anthropology*.

[17] Williams BA, Kay RF, Kirk EC (2010). New perspectives on anthropoid origins. *Proceedings of the National Academy of Sciences of the United States of America*.

[18] Beard K (1998). A new genus of Tarsiidae (Mammalia: Primates) from the middle Eocene of Shanxi Province, China, with notes on the historical biogeography of tarsiers. *Bulletin of the Carnegie Museum of Natural History*.

[19] Rasmussen DT, Conroy GC, Simons EL (1998). Tarsier-like locomotor specializations in the oligocene primate Afrotarsius. *Proceedings of the National Academy of Sciences of the United States of America*.

[20] Beard KC, Wang J (2004). The eosimiid primates (Anthropoidea) of the Heti Formation, Yuanqu Basin, Shanxi and Henan Provinces, People’s Republic of China. *Journal of Human Evolution*.

[21] Seiffert ER (2007). Early evolution and biogeography of lorisiform strepsirrhines. *American Journal of Primatology*.

[22] Yoder AD, Cartmill M, Ruvolo M, Smith K, Vilgalys R (1996). Ancient single origin for Malagasy primates. *Proceedings of the National Academy of Sciences of the United States of America*.

[23] Yoder AD, Yang Z (2004). Divergence dates for Malagasy lemurs estimated from multiple gene loci: geological and evolutionary context. *Molecular Ecology*.

[24] Karanth KP, Delefosse T, Rakotosamimanana B, Parsons TJ, Yoder AD (2005). Ancient DNA from giant extinct lemurs confirms single origin of Malagasy primates. *Proceedings of the National Academy of Sciences of the United States of America*.

[25] Godfrey LR, Jungers WL (2003). The extinct sloth lemurs of Madagascar. *Evolutionary Anthropology*.

[26] Burney DA, Burney LP, Godfrey LR (2004). A chronology for late prehistoric Madagascar. *Journal of Human Evolution*.

[27] Tornow MA (2008). Systematic analysis of the Eocene primate family Omomyidae using gnathic and postcranial data. *Bulletin of the Peabody Museum of Natural History*.

[28] Godinot M (1998). Evolution: reviewed article, a summary of adapiform systematics and phylogeny. *Folia Primatologica*.

[29] Franzen JL, Gingerich PD, Habersetzer J, Hurum JH, von Koenigswald W, Smith BH (2009). Complete primate skeleton from the middle Eocene of Messel in Germany: morphology and paleobiology. *PLoS One*.

[30] Maiolino S, Boyer DM, Bloch JI, Gilbert CC, Groenke J (2012). Evidence for a grooming claw in a North American adapiform primate: implications for anthropoid origins. *PloS One*.

[31] Marroig G, Cheverud JM (2001). A comparison of phenotypic variation and covariation patterns and the role of phylogeny, ecology, and ontogeny during cranial evolution of New World Monkeys. *Evolution*.

[32] O’Higgins P, Jones N (1998). Facial growth in *Cercocebus torquatus*: an application of three-dimensional geometric morphometric techniques to the study of morphological variation. *Journal of Anatomy*.

[33] Cardini A, Elton S (2008). Variation in guenon skulls (I): species divergence, ecological and genetic differences. *Journal of Human Evolution*.

[34] Frost SR, Marcus LF, Bookstein FL, Reddy DP, Delson E (2003). Cranial allometry, phylogeography, and systematics of large-bodied papionins (Primates: Cercopithecinae) inferred from geometric morphometric analysis of landmark data. *Anatomical Record Part A*.

[35] Lieberman DE, Ross CF, Ravosa MJ (2000). The primate cranial base: ontogeny, function, and integration. *American Journal of Physical Anthropology*.

[36] Ross CF, Kirk EC (2007). Evolution of eye size and shape in primates. *Journal of Human Evolution*.

[37] Fleagle JG, Gilbert CC, Baden AL (2010). Primate cranial diversity. *American Journal of Physical Anthropology*.

[38] Rohlf FJ, Rohlf FJ, Bookstein FL Rotational fit (Procrustes) methods.

[39] Zelditch ML, Swiderski DL, Sheets HD, Fink WL (2004). *Geometric Morphometrics for Biologists: A Primer*.

[40] Klingenberg CP (2011). MorphoJ: an integrated software package for geometric morphometrics. *Molecular Ecology Resources*.

[41] Sheets HD Integrated Morphometrics Package (IMP) 7. http://www3.canisius.edu/~sheets/imp7.htm.

